# Implications for Early Diagnosis and Treatment in Schizophrenia Due to Correlation between Auditory Perceptual Deficits and Cognitive Impairment

**DOI:** 10.3390/jcm10194557

**Published:** 2021-09-30

**Authors:** Nikolaos Moschopoulos, Ioannis Nimatoudis, Stergios Kaprinis, Kosmas Boutsikos, Christos Sidiras, Vasiliki Iliadou

**Affiliations:** 1 Clinical Psychoacoustics Lab, 3rd Psychiatry Department, School of Medicine, Faculty of Health Sciences, Aristotle University of Thessaloniki, 54636 Thessaloniki, Greece; nimatoud@med.auth.gr (I.N.); csidiras@auth.gr (C.S.); viliad@auth.gr (V.I.); 2 2nd Psychiatry Department, School of Medicine, Faculty of Health Sciences, Aristotle University of Thessaloniki, 54636 Thessaloniki, Greece; kaprinis@auth.gr (S.K.); cosmasboutsikos@gmail.com (K.B.)

**Keywords:** schizophrenia, auditory processing disorder, formal thought disorder, cognitive impairment

## Abstract

It is indicated that auditory perception deficits are present in schizophrenia and related to formal thought disorder. The purpose of the present study was to investigate the association of auditory deficits with cognitive impairment in schizophrenia. An experimental group of 50 schizophrenia patients completed a battery of auditory processing evaluation and a neuropsychological battery of tests. Correlations between neuropsychological battery scores and auditory processing scores were examined. Cognitive impairment was correlated with auditory processing deficits in schizophrenia patients. All neuropsychological test scores were significantly correlated with at least one auditory processing test score. Our findings support the coexistence of auditory processing disorder, severe cognitive impairment, and formal thought disorder in a subgroup of schizophrenia patients. This may have important implications in schizophrenia research, as well as in early diagnosis and nonpharmacological treatment of the disorder.

## 1. Introduction

Schizophrenia, despite its low prevalence, is a mental disorder that may cause significant disability [1]. How and why schizophrenia occurs is unknown. Cognitive impairment is considered a basic component of the disorder [2], while numerous studies about auditory processing deficits in patients with schizophrenia have been published. Auditory processing evaluation includes temporal processing, speech recognition, and dichotic listening (DL) testing alongside a detailed medical history as a gold-standard approach, leading to a diagnosis based on critical thinking of all tested items, as well as a consideration of comorbid conditions and related listening and communication difficulties [3,4].

The results of our recent review that examined schizophrenia studies about auditory processing [5] are noteworthy. In most cases, the only test used for auditory processing evaluation was DL, leading to incomplete evaluation of elements of auditory processing that are essential for everyday communication. Most DL studies showed that the binaural performance of patients was significantly poorer compared to that of healthy subjects [6,7,8,9,10,11]. Furthermore, patients with schizophrenia had reduced laterality [12]. A number of studies reported significant correlations between DL performance and total positive symptoms score [8,13,14]. The positive symptom of auditory hallucinations (AH) was correlated with more serious DL deficits [10,13,15,16].

Speech in noise perception is one of the most frequently used auditory processing tests, as this is one of the fundamental APD symptoms [17]. Patients with schizophrenia demonstrated poorer speech perception performance than healthy controls [18,19]. Furthermore, schizophrenia patients with auditory hallucinations exhibited worse performance in a speech perception task than patients without auditory hallucinations [20].

A number of studies have demonstrated that temporal processing is also disturbed in schizophrenia. Schizophrenia patients were less precise than healthy controls when perceiving temporal durations [21,22,23]. Moreover, Ueda et al. (2019) [24] showed in a meta-analysis that a moderate correlation exists between performance on timing tasks and the severity of positive symptoms in schizophrenia patients.

In a study by Iliadou et al. (2013) [25], patients with first episode psychosis and healthy controls completed a battery of auditory processing evaluation. It was demonstrated that patients’ performance was significantly poorer than controls in a number of tests. Nevertheless, the presence of auditory processing disorder (APD) in psychosis could not be confirmed with scientific certainty, as the individual auditory processing deficits that were discovered may not necessarily lead to APD. According to the European Consensus [26], APD is a specific deficit in the processing of auditory information along the central auditory nervous system, which includes bottom-up and top-down neural connectivity.

In a recent study [27], we implemented an auditory processing battery, which included temporal processing, speech perception, and DL tasks, to 50 schizophrenia patients and 25 healthy controls. We also investigated possible correlations between auditory processing deficits and clinical symptoms, especially the positive symptom of formal thought disorder (FTD), which was not previously examined separately. According to Çokal (2018) [28], the term “formal thought disorder” is widely used in the psychiatric literature to describe the various thought and language disturbances that clinically manifest as disorganized speech.

In brief, we discovered that [27] (1) patients with schizophrenia had significantly poorer performance than healthy controls on all auditory processing tasks, (2) the majority (64%) of schizophrenia patients were diagnosed with APD, while the vast majority (91.3%) of patients with significant FTD met the APD criteria, (3) FTD, as well as specific elements of FTD, was correlated with auditory deficits, and (4) other clinical symptoms, including delusions, were associated with auditory deficits.

In a recent letter to the editors, we outlined the design and a few of the results of the present study [29] that expand our previous findings by investigating the role of cognition in auditory processing. The interconnection between auditory processing and cognition has not previously been examined in schizophrenia, to the best of our knowledge. According to Cameron et al. (2016) [30], DL performance of children with dichotic deficits was significantly correlated with their attention and memory measures. In a recent study [31], a subgroup of individuals diagnosed with APD showed significant deficits in auditory attention.

Although auditory processing deficits and cognitive impairment are two separate conditions, they seem to overlap and interrelate. In a review paper, Boudewyn et al. (2012) [32] suggested that cognitive control deficits in schizophrenia may result in difficulties in speech processing and comprehension. There is also an indication that, in psychiatric patients, measured cognition may be poorer due to an auditory deficit, including APD [33,34].

Rönnberg et al. (2008) [35] proposed a model of speech comprehension in adverse listening conditions (e.g., noise), which stressed the role of cognitive processes. When multimodal speech information, automatically bound into a phonological representation in the episodic buffer, does not match a corresponding syllabic representation in semantic long-term memory, explicit and deliberate working memory processes are assumed to be invoked. These processes include storing of information, switching of attention, semantic integration, inference making, and inhibiting irrelevant information.

Moreover, numerous studies have demonstrated that cognitive impairment is correlated with FTD. In a meta-analysis of 52 studies, Bora et al. (2019) [36] discovered that there are significant associations between either “positive” or “negative” FTD and impairments in multiple cognitive functions, including verbal memory, visual memory, attention, processing speed, planning, fluency, and working memory. As we have already demonstrated [27] that auditory processing deficits are highly correlated with FTD in schizophrenia, the discovery of a separate association between auditory processing and cognition could be very useful in expanding our knowledge of the underlying pathophysiological processes of the disorder.

The association of auditory processing impairments with speech production abnormalities and cognitive impairment in schizophrenia is also supported by neurobiological evidence. Neural circuits responsible for auditory processing significantly interrelate and overlap with those of speech production [37,38,39,40] as well as with those of auditory attention [41], short-term verbal memory [42], and phonological working memory [43].

Gray-matter volume reductions in multiple cortical areas, including the prefrontal cortex and the superior temporal gyrus, have been identified in patients with schizophrenia [44,45,46]. Schizophrenia patients showed abnormal functional connectivity across attentional, language production, and auditory processing networks (mainly the anterior cigulate, the inferior frontal gyrus, and the superior temporal gyrus, respectively) in the resting state [47,48]. Other studies in schizophrenia found evidence of reduced functional connectivity across these regions throughout language processes, such as talking and word detection [49,50,51]. Such structural and functional brain anomalies could contribute to impairments in cognition, speech production, and auditory processing.

On the basis of the studies mentioned, we made the following prediction: auditory processing is correlated with cognition in schizophrenia patients.

## 2. Materials and Methods

### 2.1. Study Population

The study population comprised 50 schizophrenia patients, the same who participated in our previous study [27]. No participants had a history of mental retardation, neurodevelopmental disorders, traumatic head injury, neurological disorders, or a diagnosis of alcohol or substance use disorder during the last 12 months. All participants spoke Greek as a first language, and no participant was bilingual or multilingual. Subjects with a hearing loss greater than 40 dB in the better hearing ear in adults (disabling hearing loss) [52] were excluded. Nineteen subjects had hearing loss (hearing sensitivity greater than 20 dB for any of the tested frequencies), while, in nine of them, the hearing loss was unilateral and, in 10 of them, it was bilateral. All cases of hearing loss were of the sensorineural type.

The diagnosis of schizophrenia was given to the patients according to the DSM-5 [53] criteria by two experienced psychiatrists. The patients were recruited from two separate psychiatric departments, affiliated with the Aristotle University of Thessaloniki. During the time of the assessment procedure, all patients were in a stable clinical condition.

We used an empirically validated questionnaire [54] for the evaluation of the handedness of the subjects. All subjects signed informed consent. The study was approved by the Aristotle University of Thessaloniki Ethics and Bioethics Committee (date of approval: 12 July 2017, protocol number: 9. 419). Music education has been shown to enhance auditory processing [55,56]; thus, data for exposure to music education was included to be able to interpret possible outliers in the sample study. Demographic characteristics of the study population are presented in Table 1.

### 2.2. Procedure

The participants underwent an auditory processing battery of three tests. It was ensured that the given instructions were fully understood, and that the practice items of each test were successfully completed by all participants, before the initiation of the standard test procedure.

The Dichotic Digits (DD) test for the Greek language uses naturally spoken digits from 1–9 as auditory stimuli [57]. The test is composed of 20 dichotic presentations of 80 total digits (40 per ear) and also includes two practice items. Moreover, a pure tone is played as a cue before each presentation, in order to capture and sustain the attention of the participant. The DD test was repeated three times with different instructions: either to repeat all four digits, or to attend to the right or left ear digit pairs (Non-Forced (NF), Forced-Right (FR), and Forced-Left (FL) conditions). The percentage of correct responses for each ear in all three conditions was the outcome measure. In the NF paradigm, a DL laterality index was calculated, according to the following formula:

(Right-ear score − Left-ear score)/(Right-ear score + Left-ear score) × 100.(1)

According to both Bozikas et al. (2014) [6] and Hugdahl et al. (2003) [8], FR and FL conditions tap into two separate cognitive processes. In brief, the NF paradigm mostly assesses hemispheric function and asymmetry (laterality), while FR and FL conditions are assessment tools for auditory attention and top-down control of attention, respectively.

The Speech-in-Babble (SinB) test for the Greek language is a standardized test of verbal perception, developed in the Aristotle University of Thessaloniki Clinical Psychoacoustics Lab [58,59]. The test is composed of two lists (one for each ear) of 50 phonetically balanced disyllabic Greek words (such as “πνοή” [pno’i] = breath) that are presented at the same time with incomprehensible babble from multiple talkers, and it is administered monaurally. Within each list, the signal-to-noise ratio (SNR) varies. Five different SNRs (+7, +5, +3, +1, and −1) are used, beginning with the easiest listening condition (SNR +7) and ending with the most demanding listening condition (SNR −1). Each SNR is applied to 10 words in each list. The participant is instructed to repeat the word heard after each presentation. The basic Spearman–Karber formula is used to measure test performance [60,61]. Higher SNR thresholds (in dB) correspond to poorer performance.

SNR of 50% correct speech identification = i + d/2 − x/w,(2)

where i is the initial presentation level in dB, d is the step size, x is the number of items repeated correctly, and w is the number of items per step.

The Gaps-in-Noise (GIN) test [62] is an assessment tool for temporal processing. There are separate lists of 35 trials each that are administered monaurally. Before the testing procedure, participants have to complete a practice session that includes 10 trials (white noise segments). Each one of these segments consists of white noise (6 s), followed by a silence interval (5 s). Each white noise segment contains 0–3 gaps (silence intervals), the durations of which are 2, 3, 5, 6, 8, 10, 12, 15, and 20 ms. The duration and the location of gaps within the noise segments are pseudorandomized with regard to their occurrences. The participant is instructed to press a button each time they perceive a gap. Test performance is reflected by the gap detection threshold for each ear. As in the SinB test, higher thresholds (in msec) correspond to poorer performance. The gap detection threshold is the shortest gap duration for which there are at least “four of six” correct identifications.

GIN is a clinically useful test of temporal resolution. The latter reflects the perceptual threshold of a change in an auditory stimulus in the range of milliseconds and is considered to be an important component of auditory processing, as it may affect an individual’s perception of consonants during running speech [62].

All tests were delivered in a sound-treated booth via headphones (TDH-50P) through a CD player routed via a GSI 61 audiometer. Auditory stimuli were presented in each ear at 50 dB Hearing Level (HL), plus the Pure Tone Average (PTA), in order to minimize the effect of hearing threshold on auditory processing. Decibel (dB) Hearing Level (HL) is the measurement unit of the sound intensity that normalizes the different sensitivity of the human auditory system to sounds regarding the frequency. From a practical point of view, decibel (dB) Hearing Level (HL) is the unit of sound intensity that is currently used when measuring human audibility. Pure Tone Average (PTA) refers to the average of hearing threshold levels at 250, 500, 1000, 2000, 4000, and 8000 Hz.

Furthermore, a battery of four separate neuropsychological tests was administered to all participants:

Two conditions (semantic condition, phonemic condition) of the Greek Verbal Fluency (VF) task [63] for the evaluation of semantic and phonemic fluency. In the semantic condition, the participants have to produce as many words as possible from the semantic category “animals” in 60 s. In the phonemic condition, they have to produce as many words as possible from the phonemic category “words beginning with X” in 60 s.

The Digit Span (DS) task (forward and backward score) from the Wechsler Adult Intelligence Scale-III [64] for working memory and short-term memory. A sequence of numbers is presented to the participant, who has to repeat the same sequence back to the examiner in order (forward) or in reverse order (backward).

The Greek translation of the Stroop test Part 3 [65,66] for response inhibition and selective attention. A table is presented to the participants, in which color-words are printed in an ink of inconsistent color (for instance, the word “red” is printed in blue ink). The participants have to name the color of the ink instead of reading the word, as quickly as possible.

The Trail Making Test Part B (TMT-B) [67] for cognitive flexibility and processing speed. The participants are instructed to connect a set of 25 dots as fast as possible, alternating between numbers and letters (1, A, 2, B, etc.).

### 2.3. Statistical Analysis

The SPSS 25 package was used for statistical analysis. As the results did not follow a normal distribution, under the criterion of skewness and kurtosis *z*-values ranging between −1.96 and 1.96 [68,69], nonparametric tests were chosen.

A Spearman analysis was conducted to examine correlations between auditory processing scores and neuropsychological battery scores.

## 3. Results

Patients’ scores in the neuropsychological tests are presented in Table 2.

Performance in the semantic condition of the Verbal Fluency test (VF-S) was negatively correlated with GIN threshold in the right ear (rho = −0.282, *p* < 0.05).

Performance in the phonemic condition of the Verbal Fluency test (VF-P) was correlated with left-ear score in the NF condition of the DD test (rho = 0.348, *p* < 0.05), right-ear score in FR condition (rho = 0.49, *p* < 0.001), left-ear score in FL condition (rho = 0.325, *p* < 0.05), and it was negatively correlated with laterality (rho = −0.285, *p* < 0.05), left-ear score in FR condition (rho = −0.385, *p* < 0.01), and right-ear score in FL condition (rho = −0.323, *p* < 0.05). It was also negatively correlated with SNR threshold in the right ear (rho = −0.332, *p* < 0.05) and GIN threshold in the left ear (rho = −0.29, *p* < 0.05).

The Digit Span forward (DS-F) score was correlated with both right- and left-ear scores in the NF condition (rho = 0.47, *p* = 0.001; rho = 0.51, *p* < 0.001 for right and left ear, respectively), right-ear score in FR condition (rho = 0.518, *p* < 0.001), and left-ear score in FL condition (rho = 0.407, *p* < 0.01), and it was negatively correlated with laterality (rho = −0.286, *p* < 0.05), left-ear score in FR condition (rho = −0.46, *p* = 0.001), and right-ear score in FL condition (rho = −0.359, *p* = 0.01).

The Digit Span backward (DS-B) score was correlated with both right- and left-ear scores in the NF condition (rho = 0.28, *p* < 0.05; rho = 0.42, *p* < 0.01 for right and left ear, respectively), right-ear score in FR condition (rho = 0.411, *p* < 0.01), and left-ear score in FL condition (rho = 0.295, *p* < 0.05), and it was negatively correlated with laterality (rho = −0.360, *p* = 0.01) and left-ear score in FR condition (rho = −0.414, *p* < 0.01). Furthermore, it was correlated negatively with SNR threshold in the left ear (rho = −0.35, *p* < 0.05) and GIN threshold in both ears (rho = −0.415, *p* < 0.01; rho = −0.325, *p* < 0.05 for right and left ear, respectively).

Completion time in Section 3 of the Stroop test was negatively correlated with both right- and left-ear scores in the NF condition (rho = −0.464, *p* = 0.001; rho = −0.441, *p* < 0.01 for right and left ear, respectively), right-ear score in FR condition (rho = −0.308, *p* < 0.05), and left-ear score in FL condition (rho = −0.353, *p* < 0.05), and it was correlated with right-ear score in FL condition (rho = 0.324, *p* < 0.05). Furthermore, it was correlated with SNR thresholds in both ears (rho = 0.353, *p* < 0.05; rho = 0.491, *p* < 0.001 for right and left ear, respectively).

Completion time in section B of the Trail Making Test (TMT-B) was negatively correlated with left-ear score in the NF condition (rho = −0.479, *p* = 0.001) and right-ear score in FR condition (rho = −0.393, *p* = 0.01), and it was correlated with laterality (rho = 0.507, *p* = 0.001). Moreover, it was correlated with SNR thresholds in both ears (rho = 0.359, *p* < 0.05; rho = 0.315, *p* < 0.05 for right and left ear, respectively). The above results are presented in Table 3.

## 4. Discussion

In the present study, the association of auditory processing and cognition in patients with schizophrenia was investigated. Our prediction was verified, as all neuropsychological test scores were significantly correlated with at least one auditory processing test score.

VF-S scores were negatively correlated with GIN thresholds in the right ear, linking temporal resolution with verbal fluency. Moreover, significant correlations were found between VF-P scores and all auditory processing test scores. Specifically, VF-P scores were correlated with left-ear score in NF condition, right-ear score in FR condition and left-ear score in FL condition, and they were negatively correlated with laterality, left-ear scores in FR condition, right-ear scores in FL condition, SNR thresholds in the right ear, and GIN thresholds in the left ear. According to Shao et al. (2014) [70], the semantic condition of the Verbal Fluency test principally measures lexical access, while the phonemic condition mainly measures executive control. The latter can be divided into three components: updating (constant monitoring and tracking of working memory representations), shifting (flexibly switching between tasks or mental sets), and inhibition of dominant responses [70].

Lexical access, as measured by the semantic condition of the Verbal Fluency test, was correlated with temporal resolution ability in schizophrenia. Thus, a schizophrenia patient with a good consonant perception during running speech has efficient lexical access. Furthermore, the multiple correlations between executive control, as measured by the phonemic condition of the Verbal Fluency test, and performance in all auditory processing tests manifest the crucial role of executive control in processing of auditory information along the central auditory nervous system or the fact that individuals with auditory processing deficits may appear to have a degraded executive control functioning.

Patients’ performance in the DS-F condition was correlated only with DD performance in all conditions. DS-B performance was correlated with DD performance in all conditions except right ear performance in FL condition. It was also correlated with SinB left ear performance and GIN performance in both ears. DS-F is mainly used for the assessment of auditory attention, while DS-B is used to assess working memory and short-term memory [71].

In our study, auditory attention, as measured by Digit Span Forward, was correlated with performance in all conditions of DD. This result may be interpreted as dichotic listening being influenced by auditory attention, auditory attention being influenced by dichotic listening abilities, dichotic listening and auditory attention being in a two-way relation, or a third factor influencing both of them. In the current study, working memory and short-term memory, as measured by Digit Span Backward, were correlated with performance in almost all conditions of DD. This result may be interpreted as dichotic listening being influenced by memory, short-term and working memory being influenced by dichotic listening abilities, or correlation in both directions. A possible fourth explanation is that a third factor may be influencing dichotic listening, as well as working memory and short-term memory. Moreover, Digit Span Backward performance was correlated with speech in noise left ear performance and temporal resolution, as measured by GIN. This association links temporal resolution, speech in noise performance, and short-term and working memory. It might be inferred that strengthening one of these factors through auditory training may positively influence the others.

The completion time of the third section of the Stroop test was correlated negatively with DD scores in all conditions, except right-ear score in the FL condition. It was also correlated with SNR thresholds in both ears. This section is considered to measure the ability to inhibit cognitive interference, as well as other cognitive functions such as working memory, processing speed, attention, and cognitive flexibility [72,73].

It should be noted that there was a significant correlation between patients’ performance in the third section of the Stroop test and their performance in FL condition of the DD test. The response inhibition ability, concerning visual stimuli, is associated with the top-down control of attention, concerning auditory stimuli. The results show that these processes have similar and complementary roles in schizophrenia. It appears that more basic sensory abilities and deficits influence higher-order cognitive functions.

The completion time of the Trail Making Test section B was negatively correlated with DD left-ear scores in NF condition and right-ear scores in FR condition. Moreover, it was correlated with laterality and SinB thresholds in both ears. In addition to processing speed and cognitive flexibility, TMT-B measures executive control, which is necessary to manage switches between two different sequences [74].

More positive laterality indices (right-oriented) and better SinB thresholds were correlated with better performance in Trail Making Test Section, pinpointing to a link between speech perception in the real world and executive control related to vision.

**Figure 1:**
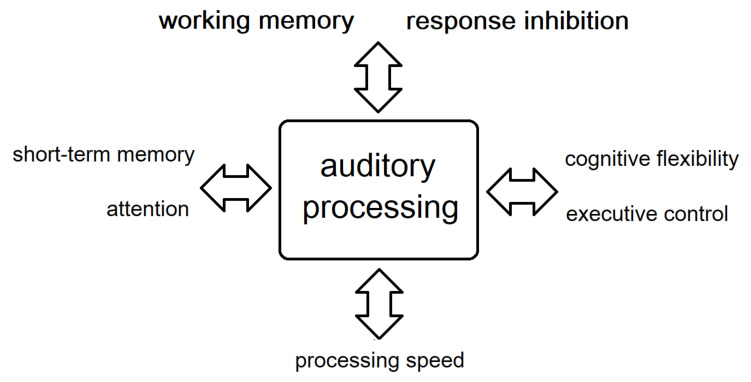
Correlations between auditory processing and cognitive functions.

Although we examined auditory processing and cognitive functions as independent processes, they seem to be quite interdependent. This pattern of results is congruent with the model of Rönnberg et al. (2008) [35], which focused on the involvement of working memory processes in speech comprehension in adverse conditions. Our results suggest that deficits in numerous cognitive processes, in addition to working memory, coexist and correlate with auditory processing deficits in schizophrenia patients. In particular, working memory and response inhibition, as well as short-term memory, attention, processing speed, cognitive flexibility, and executive control, were correlated with auditory processing performance. This novel finding, combined with the recently discovered correlation between auditory processing deficits and FTD in schizophrenia [27] could have important implications in schizophrenia research. The above correlations are presented in Figure 1.

**Figure 2:**
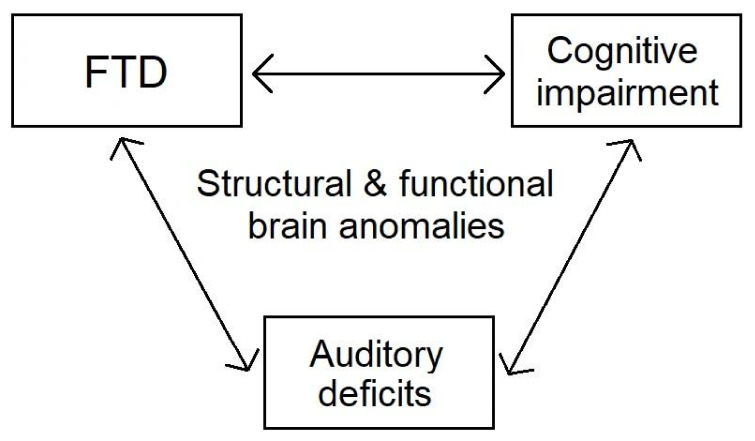
Interrelations of FTD, cognitive impairment, and auditory deficits in schizophrenia.

As mentioned in Section 1, patients with schizophrenia exhibit structural changes across multiple brain regions, including temporal and prefrontal areas, which play an important role in auditory processing, speech production, and neurocognition. Moreover, they show abnormal functional connectivity across these regions. Such brain anomalies likely set the stage for disruptions in speech perception, speech production, and neurocognition, which interrelate in schizophrenia. The above associations are presented in Figure 2. A more systematic approach to these processes would be beneficial in understanding the complex pathophysiology of schizophrenia, as well as in ameliorating the patients’ quality of life.

We made a hypothesis [5] that individuals with schizophrenia could be divided into various subgroups, which may differ in their auditory processing abilities, their neurocognition, and their clinical symptoms. The results of the present study support our hypothesis.

Auditory processing disorder may have detrimental effects on the affected individuals, interfering with emotional, communicational, social, and academic/work aspects of life, while making community inclusion more difficult [26]. Schizophrenia patients with APD and severe FTD, as well as cognitive impairment, could face even greater difficulties in communicating effectively with others. Focusing on this particular patient subgroup (APD, severe FTD, and cognitive impairment) would lead to additional management approaches with individualized auditory training, which could significantly improve the patients’ auditory processing, neurocognition, and functionality [75,76,77].

Our findings also have important clinical implications for people at high risk of developing psychosis. According to the North American Prodrome Longitudinal Study [78], impairments in declarative and working memory, as well as attention, combined with the presence of attenuated positive symptoms, could predict transition to psychosis in individuals at Clinical High Risk (CHR) stage. In the present study it was demonstrated that auditory processing deficits are associated with impairments in attention, working memory, and short-term memory. Therefore, the value of some low-cost and brief tests, such as DD, SiB, and GIN, as predictors to psychotic transition may be considered in future studies.

CHR individuals with diagnosed auditory processing deficits could also be given the option of nonpharmacological, high-benefit and low-risk interventions [79]. According to Loewy et al. (2016) [80], CHR individuals showed an improvement in verbal memory, after completing an auditory processing-based training program. Verbal memory is considered a domain that predicts later functioning, as well as psychotic transition [80].

An important limitation of the present study should be noted. Our patient sample consisted of stabilized patients; thus, it may not be representative of the entire schizophrenia population. However, extremely disorganized patients could have significant difficulties in understanding the instructions of the several tests that were administered, as well as in completing the tests.

## 5. Conclusions

The present study provides clear evidence that auditory processing deficits in schizophrenia are associated with impairment in numerous cognitive functions, such as working memory, short-term memory, attention, response inhibition, cognitive flexibility, processing speed, and executive control. Future research in the field of auditory processing in schizophrenia could contribute to the earlier diagnosis and more effective treatment of this severe and disabling mental disorder.

## Figures and Tables

**Table 1:** Demographic characteristics of participants.

	Mean	SD
Age (range)	39.18 (18–62)	10.817
Sex (male/female)	29/21	
Handedness (right/left/mixed)	39/4/7	
Education	14.0	2.886
Music education	0.92	1.794

Note: education, years of education; music education, years of music education.

**Table 2:** Neuropsychological battery scores.

Tests	*n*	Min	Max	Mean	SD
VF-S	50	7	25	15.96	4.323
VF-P	50	2	15	8.20	3.017
DS-F	50	5	13	7.78	1.569
DS-B	50	2	9	5.24	1.661
Stroop_3	49	75	253	145.69	44.803
TMT-B	42	56	268	130.48	62.345

Note: *n*, number of participants that completed the test; min, minimum value; max, maximum value; SD, standard deviation; VP-S, Verbal Fluency Semantic condition; VF-P, Verbal Fluency Phonemic condition; DS-F, Digit Span Forward; DS-B, Digit Span Backward; Stroop_3, Stroop Section 3 completion time; TMT-B, Trail Making Test section B completion time.

**Table 3:** Correlations between auditory processing scores and neuropsychological battery scores.

		VF-S	VF-P	DS-F	DS-B	Stroop_3	TMT-B
NF_RE	rho	−0.015	0.160	0.470 **	0.280 *	−0.464 **	−0.146
	Sig. (2-tailed)	0.915	0.266	0.001	0.049	0.001	0.358
NF_LE	rho	0.128	0.348 *	0.510 **	0.420 **	−0.441 **	−0.479 **
	Sig. (2-tailed)	0.377	0.013	0.000	0.002	0.002	0.001
Laterality	rho	−0.080	−0.285 *	−0.286 *	−0.360 *	0.187	0.507 **
	Sig. (2-tailed)	0.581	0.045	0.044	0.010	0.199	0.001
FR_RE	rho	0.097	0.490 **	0.518 **	0.411 **	−0.308 *	−0.393 **
	Sig. (2-tailed)	0.504	0.000	0.000	0.003	0.031	0.010
FR_LE	rho	−0.043	−0.385 **	−0.460 **	−0.414 **	0.206	0.289
	Sig. (2-tailed)	0.769	0.006	0.001	0.003	0.155	0.064
FL_RE	rho	−0.080	−0.323 *	−0.359 *	−0.263	0.324 *	0.082
	Sig. (2-tailed)	0.578	0.022	0.010	0.065	0.023	0.604
FL_LE	rho	0.081	0.325 *	0.407 **	0.295 *	−0.353 *	−0.160
	Sig. (2-tailed)	0.576	0.021	0.003	0.037	0.013	0.312
SNR_RE	rho	−0.222	−0.332 *	−0.146	−0.262	0.353 *	0.359 *
	Sig. (2-tailed)	0.121	0.019	0.312	0.066	0.013	0.020
SNR_LE	rho	−0.220	−0.268	−0.279	−0.0350 *	0.491 **	0.315 *
	Sig. (2-tailed)	0.124	0.060	0.050	0.013	0.000	0.042
GIN_RE	rho	−0.282 *	−0.274	−0.254	−0.415 **	0.218	0.209
	Sig. (2-tailed)	0.047	0.054	0.075	0.003	0.132	0.183
GIN_LE	rho	−0.236	−0.290 *	−0.130	−0.325 *	0.278	0.301
	Sig. (2-tailed)	0.099	0.041	0.368	0.021	0.053	0.053

Note: NF, Non-Forced condition of the Dichotic Digits test; RE, right ear; LE, left ear; FR, Forced-Right condition of the Dichotic Digits test; FL, Forced-Left condition of the Dichotic Digits test; SNR, SNR threshold (50% correct word identification); GIN, Gaps-in-Noise threshold in ms; VF-S, Verbal Fluency Semantic condition; VF-P, Verbal Fluency Phonemic condition; DS-F, Digit Span Forward; DS-B, Digit Span Backward; Stroop_3, Stroop Section 3 completion time; TMT-B, Trail Making Test section B completion time; ** correlation is significant at the 0.01 level (two-tailed); * correlation is significant at the 0.05 level (two-tailed).
